# Thoracic SMARCA4‐deficient undifferentiated tumor diagnosed by transbronchial mediastinal cryobiopsy: A case report

**DOI:** 10.1111/1759-7714.14830

**Published:** 2023-02-24

**Authors:** Chihiro Takemura, Tatsuya Imabayashi, Hideaki Furuse, Keigo Uchimura, Yuji Matsumoto, Takaaki Tsuchida, Shun‐ichi Watanabe

**Affiliations:** ^1^ National Cancer Center Japan, Respiratory Endoscopy Division Chuo‐ku Japan; ^2^ Department of Endoscopy, Respiratory Endoscopy Division National Cancer Center Hospital Chuo‐ku Tokyo Japan; ^3^ Department of Endoscopy, Respiratory Endoscopy Division National Cancer Center Japan Chuo‐ku Tokyo Japan; ^4^ Respiratory Endoscopy Division National Cancer Center Japan Chuo‐ku Tokyo Japan; ^5^ Department of Thoracic Oncology National Cancer Center Japan Chuo‐ku Japan; ^6^ Division of Thoracic Surgery National Cancer Center Hospital Chuo‐ku Tokyo Japan

**Keywords:** bronchoscopy, cryobiopsy, endobronchial ultrasound‐guided transbronchial needle aspiration, lung cancer, next‐generation sequencing

## Abstract

Thoracic SMARCA4‐deficient undifferentiated tumors (SMARCA4‐UT) have a poor prognosis and are often diagnosed at an inoperable advanced stage. Herein, we report a case of SMARCA4‐UT diagnosed by endobronchial ultrasound‐guided transbronchial cryobiopsy (EBUS‐cryo). The patient was a 42‐year‐old man with a history of smoking. Chest computed tomography revealed a right upper lobe nodule and an enlarged #11s lymph node. Core tissues could not be obtained by EBUS‐guided transbronchial needle aspiration (EBUS‐TBNA) for diagnosis and mediastinal staging; hence, EBUS‐guided intranodal forceps biopsy (EBUS‐IFB) was performed. However, a detailed diagnosis beyond poorly differentiated carcinoma could not be obtained. Subsequent EBUS‐cryo provided sufficient specimens for immunohistochemical and molecular evaluation and SMARCA4‐UT was definitively diagnosed. Thus, EBUS‐cryo could be of additional diagnostic value for uncommon tumors, such as SMARCA4‐UT, conjointly with EBUS‐IFB as well as EBUS‐TBNA.

## Graphical abstract

We report a case of SMARCA4‐UT that was successfully diagnosed via EBUS‐cryo after unsuccessful EBUS‐TBNA and EBUS‐IFB. Conventional endobronchial ultrasonography‐guided procedures cannot provide sufficient biopsy samples for the diagnosis and staging of rare mediastinal tumors. Endobronchial ultrasonography‐guided transbronchial cryobiopsy helps obtain adequate tissue samples for histological, immunohistochemical, and molecular (including next‐generation sequencing) evaluations.

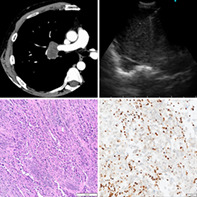

## INTRODUCTION

Thoracic SMARCA4‐deficient undifferentiated tumor (SMARCA4‐UT) is a high‐grade malignancy, often affecting young adults with heavy smoking history and emphysema.^1^ Distinguishing SMARCA4‐UT from poorly differentiated lung cancer can be difficult, especially in small biopsy specimens.^2^ However, careful clinicopathological assessment can provide a definitive diagnosis.^1^

Endobronchial ultrasound‐guided transbronchial needle aspiration (EBUS‐TBNA) is a well‐established method for diagnosing mediastinal and hilar lesions.^3^ However, core tissues obtained by EBUS‐TBNA are sometimes inadequate for immunostaining because of tissue volume, blood contamination, and crushing.^4^, ^5^ Recently, EBUS‐guided transbronchial mediastinal cryobiopsy (EBUS‐cryo), which can obtain larger high‐quality tissue samples with fewer crush artifacts, has demonstrated advantages over EBUS‐TBNA in diagnosing uncommon mediastinal tumors.^6^, ^7^, ^8^, ^9^, ^10^ However, combined EBUS‐cryo and EBUS‐guided intranodal forceps biopsy (EBUS‐IFB) remain to be reported.^5^

Herein, we report a case of SMARCA4‐UT successfully diagnosed by adding EBUS‐cryo after EBUS‐TBNA and EBUS‐IFB.

## CASE REPORT

**FIGURE 1:**
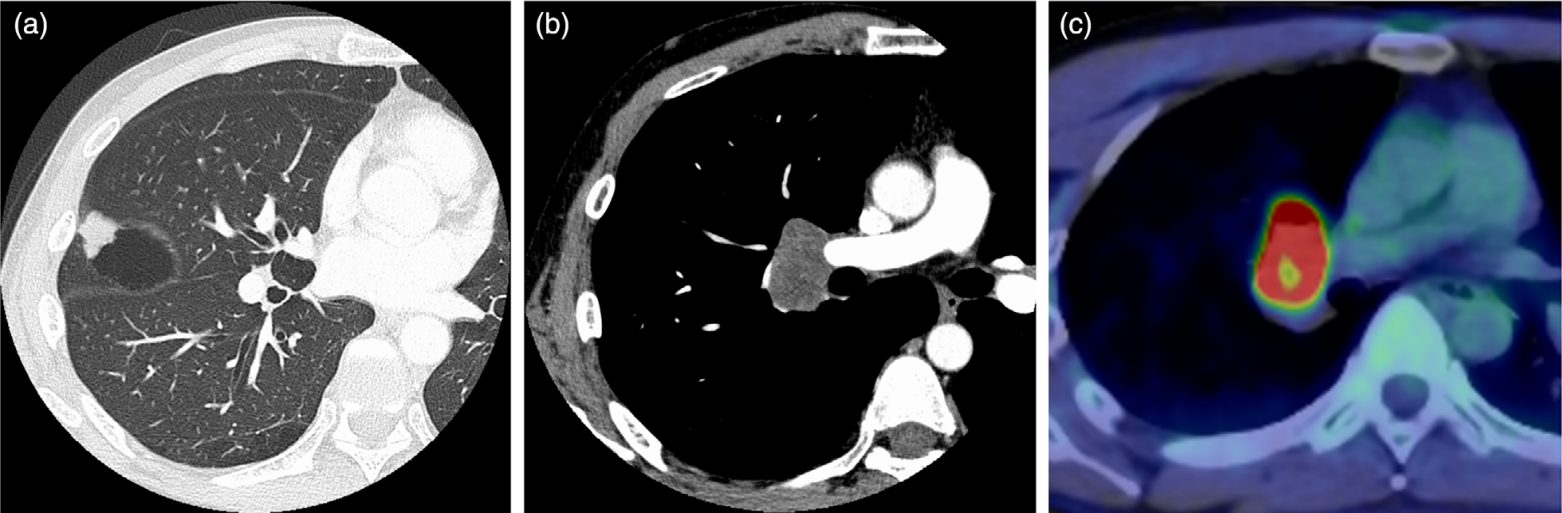
Chest computed tomography (CT) and positron emission tomography (PET)‐CT findings. (a, b) Chest CT revealed a 1.6‐cm‐sized nodule with a pulmonary cyst in the interlobar pleura of right upper and middle lobes and an enlarged #11s lymph node. (c) The lymph node exhibited fluorodeoxyglucose uptake with a maximum standardized uptake value of 17.78 on PET‐CT.

**FIGURE 2:**
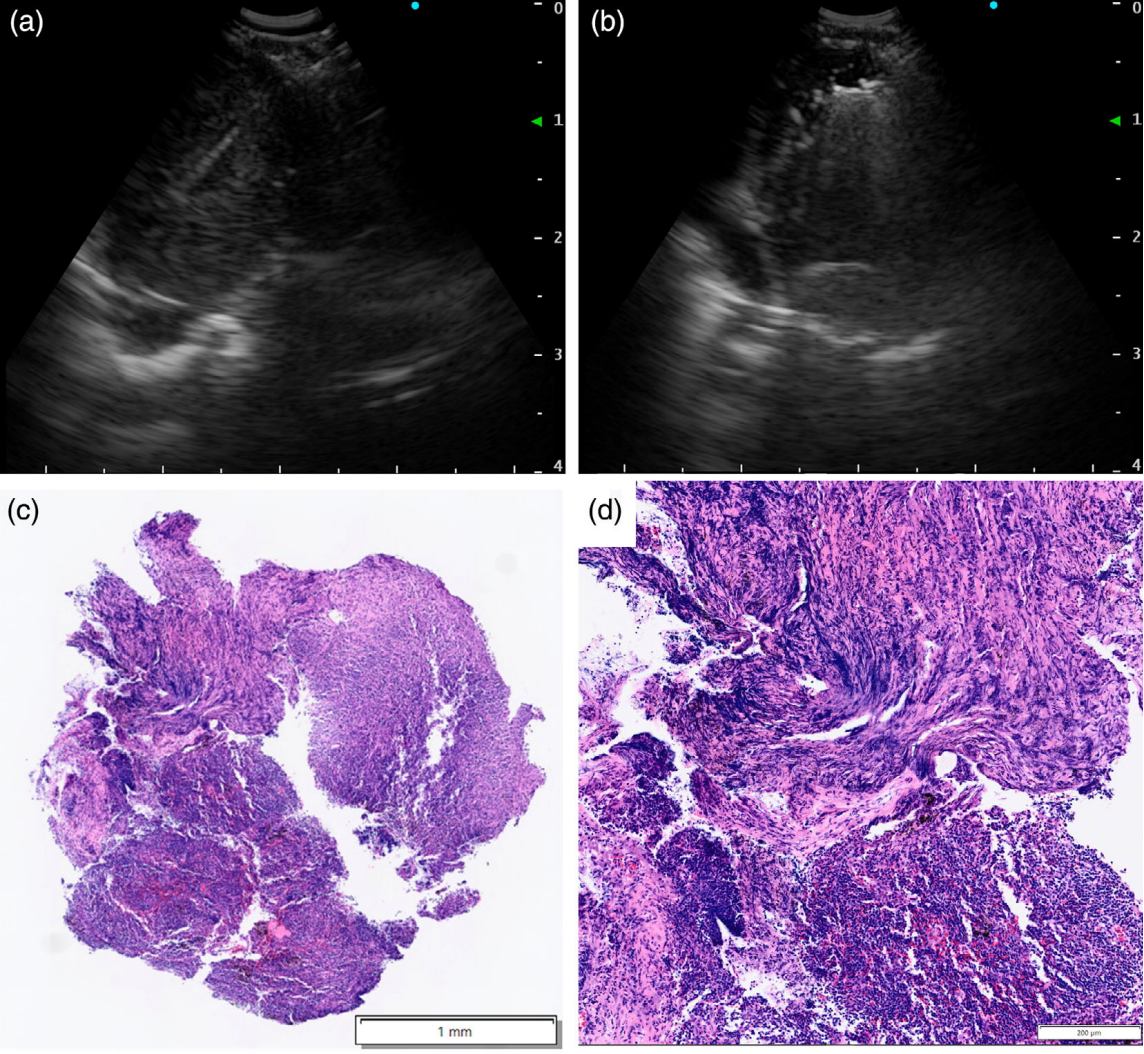
Endobronchial ultrasound (EBUS) images and pathological findings of the EBUS‐guided intranodal forceps biopsy (EBUS‐IFB) specimen. (a) An EBUS image depicting the EBUS‐guided transbronchial needle aspiration (EBUS‐TBNA) for the enlarged #11s lymph node. The TBNA samples, although showing positive finding for malignancy by rapid on‐site assessment, could not be submitted as core tissue specimens, owing to the predominance of necrotic fluid component. (b) An EBUS image depicting the EBUS‐IFB of the #11s lymph node. In the modified EBUS‐IFB technique, it is technically easier to create puncture holes against flat surfaces, such as #4R and #7, than against pointed surfaces between a bifurcation, such as #11s. (c, d) Hematoxylin and eosin staining of the EBUS‐IFB specimen collected with 1.9‐mm forceps. The area of the specimen is 4.55 mm^2^. It exhibits a sheet of undifferentiated round cells with prominent nucleoli, which are markedly accompanied by crush artifacts caused by forceps compression and inflammatory cell infiltration.

**FIGURE 3:**
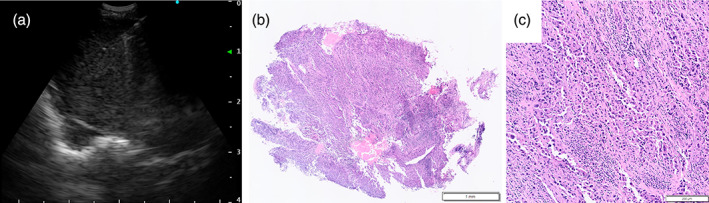
Endobronchial ultrasound (EBUS) image depicting the EBUS‐guided transbronchial mediastinal cryobiopsy (EBUS‐cryo) for #11s lymph node and pathological findings of the specimen. (a) EBUS‐cryo is performed using a 1.7‐mm cryoprobe with freezing time of 4 s. (b, c) Hematoxylin and eosin staining of the EBUS‐ cryospecimen. The area of the specimen is 9.81 mm^2^. It exhibits proliferation of giant cells and large cells, which are loosely connected and poorly differentiated.

**FIGURE 4:**
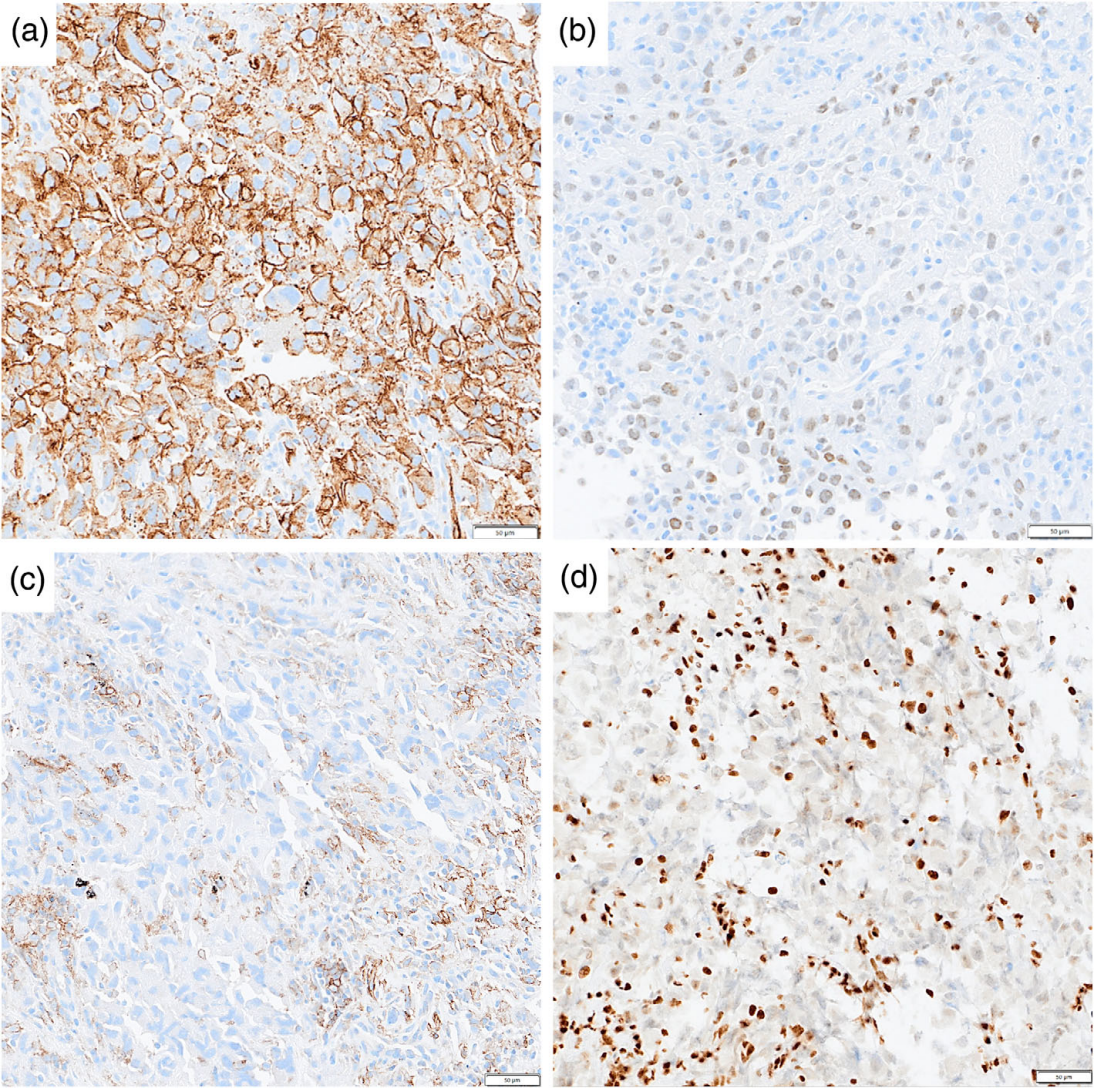
Immunohistochemical staining of a transbronchial mediastinal cryobiopsy specimen. (a) Diffuse expression of CD34. (b) Partially positive expression of SALL4. (c) Reduction and loss of SMARCA4 in tumor cells. (d) The programmed death ligand‐1 tumor proportion score using the 22C3 pharmDX assay is 15%.

A 42‐year‐old man, a current smoker (22 pack‐years), was referred for a right hilar mass. Blood tests showed no abnormal carcinoembryonic antigen, cytokeratin 19 fragment, or progastrin‐releasing peptide levels. Chest computed tomography revealed a nodule and a pulmonary cyst between the right upper and middle lobes and an enlarged #11s lymph node, which revealed an abnormal uptake of fluorodeoxyglucose on positron emission tomography (Figure 1a–c). The #11s lymph node was punctured thrice with a 25‐gage needle (NA‐U401SX‐4025 N, Olympus) (Figure 2a) during EBUS‐TBNA for diagnosis and staging (#4R and #7 metastases were ruled out). Since no core tissue was obtained, an additional four specimens were obtained with 1.9‐mm forceps (FB‐231‐D, Olympus) using the modified EBUS‐IFB technique (Figure 2b).^11^ The EBUS‐IFB specimens were markedly degenerative with inflammatory cell infiltration, and a detailed diagnosis beyond poorly differentiated carcinoma (Figure 2c,d) could not be obtained. The carcinoma was diagnosed as clinical T1bN1M0 stage IIB; however, the patient experienced a stroke preoperatively. Given his deteriorating general condition, EBUS‐cryo was performed for detailed histological diagnosis. A needle tract was created by EBUS‐TBNA using the aforementioned modified EBUS‐IFB technique, and EBUS‐cryo was performed four times with a 1.7‐mm cryoprobe (20402–410, ERBE) (Figure 3a). The EBUS‐cryo specimens revealed proliferation of giant and large cells, which were loosely connected and poorly differentiated (Figure 3b,c). Immunohistochemically, the tumor demonstrated reduced and lost staining for SMARCA4 expression; it was negative for cytokeratin (AE1/3 and CK‐OSCAR) and S100, positive for CD34, and partially positive for SALL4 (Figure 4a–d). Consequently, the patient was diagnosed with SMARCA4‐UT with no targetable driver mutations using the Oncomine Dx Target Test multi‐CDx system (Thermo Fisher Scientific) and a programmed death ligand‐1 tumor proportion score of 15%. Owing to SMARCA4‐UT's insensitivity to chemotherapy, surgery was performed after his general condition improved. No postoperative adjuvant therapy was administered and no recurrence has occurred during the last 3 months.

## DISCUSSION

In our case, EBUS‐cryo provided sufficient specimens for immunohistochemical and molecular evaluation to help determine the treatment strategy for resectable SMARCA4‐UT. Mutations in *STK11* and *KEAP1* have been implicated for resistance to immunotherapy.^12^ Next‐generation sequencing (NGS) analysis of these mutations may be important in expanding the limited treatment options for SMARCA4‐UT.

A recent randomized controlled trial demonstrated the additional diagnostic value of EBUS‐cryo over EBUS‐TBNA.^9^ Regarding sample quality and quantity, no studies have compared the diagnostic performance of EBUS‐IFB and EBUS‐cryo; however, EBUS‐cryo may be superior to EBUS‐IFB, as in our case. Furthermore, EBUS‐cryo may also be advantageous in NGS analysis. In transbronchial lung biopsies, the amounts of DNA and RNA extracted from cryobiopsy samples were reported to be approximately three times more than those extracted from forceps biopsy samples.^13^

A meta‐analysis of 21 studies including 1175 patients reported that the pooled proportion of adequate EBUS‐TBNA samples for NGS was 86.5%.^14^ Moreover, the NGS success rate reportedly increased as the number of passes or core tissues obtained increased and also did not change with needle size (22 or 25‐gauge).^15^ If, as in our case, core tissue cannot be collected despite a positive finding for malignancy on rapid on‐site evaluation, EBUS‐cryo can complement EBUS‐TBNA, except in settings where cytology specimens are available for NGS. Based on the rate of core tissue obtained per pass, approximately 60%,^16^ the range of indications for EBUS‐cryo in clinical practice may be wider than expected.

The primary technical challenge of EBUS‐cryo is to establish a standard technique for creating a cryoprobe insertion tract into the lymph node. The high success rate of a high‐frequency needle‐knife incision technique has been demonstrated in two large randomized controlled trials.^7^, ^9^ In our case, the puncture hole was widened by tunneling with the needle alone using a combination of angulation and rotational maneuvers.^11^ This modified EBUS‐IFB technique has also demonstrated a high technical IFB success rate of 90.8% (177/195). Other reported techniques include widening the puncture hole by advancing the needle sheath^8^ or establishing a working channel for EBUS‐cryo by advancing the sheath into the lesion.^17^ The main complications of these techniques are post‐procedural infection and pneumothorax in up to 1% and minor bleeding in approximately 10% of patients, with no apparent increased risk compared with EBUS‐TBNA alone. EBUS‐cryo may be a good indication when EBUS‐TBNA does not provide sufficient samples. However, future studies are needed to determine which technique is superior, and whether to choose EBUS‐cryo or EBUS‐IFB after considering their costs.

In summary, we report a case of SMARCA4‐UT diagnosed by EBUS‐cryo. Thus, EBUS‐cryo may provide additional diagnostic value to EBUS‐IFB and EBUS‐TBNA for the histological and molecular evaluation of mediastinal and hilar lesions.

## AUTHOR CONTRIBUTIONS

All authors read and approved the final manuscript. *Writing – Original Draft*: Chihiro Takemura. *Patient Care*: Tatsuya Imabayashi, Hideaki Furuse, Keigo Uchimura, Yuji Matsumoto, Shun‐ichi Watanabe, and Takaaki Tsuchida. *Writing ‐ Review & Editing*: Tatsuya Imabayashi, Hideaki Furuse, Keigo Uchimura, Yuji Matsumoto, Shun‐ichi Watanabe, and Takaaki Tsuchida.

## CONFLICT OF INTEREST

The authors declare that they have no competing interests.
